# The untapped potential of targeting NRF2 in neurodegenerative disease

**DOI:** 10.3389/fragi.2023.1270838

**Published:** 2023-09-28

**Authors:** Wei-Tai Chen, Matthew Dodson

**Affiliations:** Department of Pharmacology and Toxicology, College of Pharmacy, University of Arizona, Tucson, AZ, United States

**Keywords:** Nrf2, therapeutics, neurodegeneration, KEAP1, oxidative stress

## Abstract

Since its initial discovery almost three decades ago, the transcription factor nuclear factor erythroid 2-related factor 2 (NRF2) has been shown to regulate a host of downstream transcriptional responses and play a critical role in preventing or promoting disease progression depending on the context. Critically, while the importance of proper nuclear factor erythroid 2-related factor 2 function has been demonstrated across a variety of pathological settings, the ability to progress NRF2-targeted therapeutics to clinic has remained frustratingly elusive. This is particularly true in the case of age-related pathologies, where nuclear factor erythroid 2-related factor 2 is a well-established mitigator of many of the observed pathogenic effects, yet options to target this pathway remain limited. Along these lines, loss of nuclear factor erythroid 2-related factor 2 function has clearly been shown to enhance neuropathological outcomes, with enhancing nuclear factor erythroid 2-related factor 2 pathway activation to prevent neurodegenerative/neurological disease progression continuing to be an active area of interest. One critical obstacle in generating successful therapeutics for brain-related pathologies is the ability of the compound to cross the blood brain barrier (BBB), which has also hampered the implementation of several promising nuclear factor erythroid 2-related factor 2 inducers. Another limitation is that many nuclear factor erythroid 2-related factor 2 activators have undesirable off-target effects due to their electrophilic nature. Despite these constraints, the field has continued to evolve, and several viable means of targeting nuclear factor erythroid 2-related factor 2 in a neuropathological context have emerged. In this perspective, we will briefly discuss the key findings and promising therapeutic options that have been discovered to date, as well as highlight emerging areas of NRF2-neurodegeneration research that provide hope for successfully targeting this pathway in the future.

## Introduction

The transcription factor nuclear factor erythroid 2-related factor 2 (NRF2) is a critical regulator of cell survival, mediating vital aspects of redox, protein, and metabolic homeostasis. In fact, NRF2 target genes are involved in glutathione synthesis, peroxide reduction, xenobiotic detoxification, proteasome assembly, autophagy, transport and storage of iron, lipid catabolism, and carbohydrate metabolism (Dodson et al., 2019). Accordingly, dysregulation of NRF2 signaling has been linked to promoting disease progression, with restoration of proper NRF2 function restoring homeostasis (Cuadrado et al., 2019). Perhaps one of the greatest ongoing frustrations in the field is that despite an ever-increasing number of compounds that beneficially activate or inhibit NRF2 in an experimental setting, very few have progressed through clinical trials to become viable therapeutics. This shortfall also holds true for neurodegenerative diseases, where loss of NRF2 is a well-established driver of neurodegenerative phenotypes, yet restoration of NRF2 signaling remains an unharnessed therapeutic possibility.

Critically, NRF2 has been shown to play an important role in mitigating the onset and progression of several neurodegenerative diseases, including Parkinson’s disease (PD), Alzheimer’s disease (AD), Huntington’s disease (HD), and Multiple sclerosis (MS) (Cuadrado, 2022). Loss of NRF2 function significantly exacerbates neurodegenerative phenotypes, often resulting in increased inflammation, oxidative stress, or proteotoxicity that enhance pathogenesis of the chosen model (Brandes and Gray, 2020). Importantly, NRF2 activity has been shown to decline with age (Suh et al., 2004; Zhou et al., 2018), inferring that the greatest risk factor for developing neurodegenerative disease is associated with progressive loss of NRF2. This indicates that restoring proper NRF2 function, either by direct activation of NRF2 or blocking the mechanisms that leads to its decline, represents a feasible strategy to prevent onset and progression of these debilitating diseases. Below, we will give a brief overview of the NRF2 signaling pathway and discuss the experimental evidence supporting a role for NRF2 across different neurodegenerative contexts. Next, we will highlight the compounds identified to date that have shown the most therapeutic promise, as well as the feasibility of utilizing gene therapy-based approaches and drug delivery systems to achieve a more potent and targeted effect. Finally, we will discuss the future of the NRF2-aging field, including the key barriers that need to be overcome to progress the science from experimental evidence to actual translational applications.

### Nrf2 and neurodegeneration

#### The Nrf2 signaling pathway

NRF2 is regulated primarily at the protein level by the Kelch-like ECH-associated protein 1-Cullin-3-RING box protein-1 (KEAP1-CUL3-RBX1) E3 ubiquitin ligase complex. Under basal, non-stressed conditions, NRF2 is targeted by this complex for proteasomal degradation (Itoh et al., 1999; Kobayashi et al., 2004; Zhang et al., 2004); however, upon the introduction of electrophilic/oxidative stress (Dinkova-Kostova et al., 2002; Zhang and Hannink, 2003), mutations in NRF2 or its degradation machinery (Singh et al., 2006; Shibata et al., 2008; Martinez et al., 2013; Ooi et al., 2013), or autophagy dysfunction (Komatsu et al., 2010; Lau et al., 2010), NRF2 accumulates in the nucleus and binds small musculoaponeurotic fibrosarcoma F/G/K (sMaf F/G/K) proteins to activate transcription of antioxidant response element (ARE)-containing target genes (Itoh et al., 1997). Along with KEAP1-dependent degradation, glycogen synthase kinase β (GSK3-β)-dependent phosphorylation of NRF2 can result in recruitment of the S-phase kinase-associated protein 1-Cullin-1-Rbx1/β-transducin repeat-containing protein (SCF/β-TrCP) E3 complex and degradation of NRF2 (Rada et al., 2011). Additionally, synoviolin-1 (also known as Hrd1) is an E3 ligase that has been shown to degrade NRF2 in the endoplasmic reticulum, particularly during liver cirrhosis (Wu et al., 2014). Along with ubiquitination, other posttranslational modifications, including acetylation (Sun et al., 2009), phosphorylation (Huang et al., 2002), methylation (Liu et al., 2016), and SUMOylation (Malloy et al., 2013) of NRF2, as well as OGlcNAcylation of KEAP1 (Chen et al., 2017), have been shown to dictate NRF2 localization and stability. It is also worth noting that NRF2 expression can be regulated at the DNA and mRNA levels. For example, methylation of the *KEAP1* or *NFE2L2*/NRF2 promoters (Wang et al., 2008; Muscarella et al., 2011; Khor et al., 2014), as well as transcriptional up or downregulation of NRF2 expression by other transcription factors (i.e., nuclear factor kappa B [NF-κB] and aryl hydrocarbon receptor [AhR]) (microRNA) (Miao et al., 2005; Liu et al., 2008), have all been shown to dictate the NRF2 response. Finally, several microRNAs (i.e., miR-27a, miR144, miR153, and miR142-5p) have been reported to suppress expression of NRF2 (Narasimhan et al., 2012; Zhao et al., 2018; Chu et al., 2019). Overall, it is clear why NRF2 dysregulation leads to disease, as the complex and interconnected nature of the NRF2 signaling cascade presents a multitude of possible points of dysfunction. Below, we will highlight evidence of NRF2 importance in the context of different age-related neurodegenerative disorders.

#### Parkinson’s disease

**FIGURE 1:**
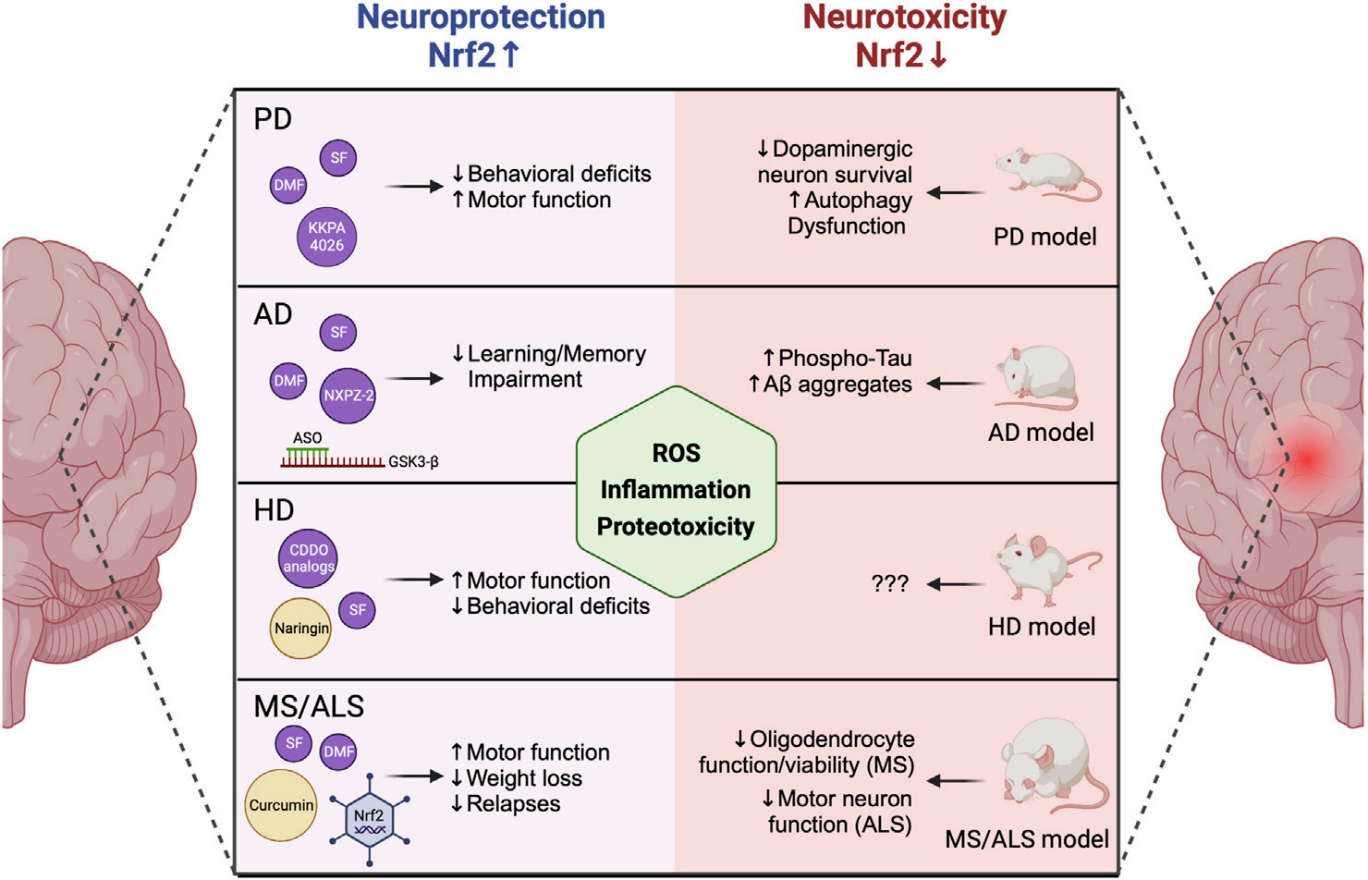
NRF2 levels mediate neurodegenerative disease progression. NRF2 activation via covalent and non-covalent pharmacological modifiers or genetic modulation of KEAP1 has been linked to decreased behavioral deficits, increased motor function, decreased learning and memory impairment, and decreased weight loss and disease relapse in animal models of PD, AD, HD, and MS/ALS. Contrastingly, loss of NRF2 enhances neurodegenerative phenotypes, including increased ROS-, inflammation-, and proteotoxicity-dependent inhibition of neuronal and glial function and viability. PD = Parkinson’s disease, AD = Alzheimer’s disease, HD = Huntington’s disease, MS = Multiple sclerosis, ALS = Amyotrophic lateral sclerosis, ROS = Reactive oxygen species. Created with Biorender.

Several experimental studies have demonstrated the importance of NRF2 in preventing the development of PD phenotypes. For example, on the chemical induction front, numerous studies utilized administration of 1-methyl-4-phenyl-1,2,3,6-tetrahydropyridine (MPTP), a well-established chemical inducer of dopaminergic neuron death and the onset of parkinsonian phenotypes, with *Nrf2* ^*−/−*^ mice exhibiting a more pronounced loss of dopaminergic function and onset of PD-related phenotypes (Burton et al., 2006; Chen et al., 2009; Innamorato et al., 2010; Rojo et al., 2010; Kaidery et al., 2013). Importantly, similar results were obtained with rotenone, 6-hydroxydopamine, and paraquat, three other notable chemical inducers of PD-relevant outcomes (Jakel et al., 2007; Wang et al., 2017; Wei et al., 2020). Supporting this notion, a series of recent studies from us and a collaborator’s group showed that loss of NRF2 significantly enhanced dopaminergic neuron loss, autophagy dysfunction, inflammation, and cell death in the Thy1 mouse model of human α-synuclein overexpression (Corenblum et al., 2016; Ray et al., 2018; Anandhan et al., 2021; Anandhan et al., 2022). A similar study indicated that *Nrf2* ^*−/−*^ mice were more susceptible to PD phenotypes when stereotactically injected with adenoviral α-synuclein (Lastres-Becker et al., 2012). This infers that both genetic and chemical models of PD are enhanced by genetic ablation of NRF2, clearly indicating its importance in preventing disease progression (summarized in Figure 1, right panel).

Conversely, several studies have shown that genetic ablation/suppression of *KEAP1*, which results in constitutive upregulation of the NRF2 signaling cascade, improves neuronal survival and decreases PD phenotypes both *in vitro* and *in vivo* (Satoh et al., 2009; Williamson et al., 2012). Interestingly, specific overexpression of *NFE2L2/*NRF2 in astrocytes was shown to prevent MPTP-induced PD pathogenesis (Chen et al., 2009). Thus, an ongoing area of interest in the field is determining the cell type-specific relevance of NRF2 in glia (i.e., astrocytes, oligodendrocytes, and microglia) versus neurons in dictating PD progression (Liddell, 2017), with much work still to be done. Much like genetic modification of *KEAP1* or *NFE2L2/*NRF2 itself, several NRF2 inducers have been shown to ameliorate pathogenic features of PD. This includes covalent (i.e., sulforaphane [SF], di-/mono-methyl fumarate [DMF/MMF], bardoxolone analogs [CDDO], and curcumin), and non-covalent (i.e., KKPA4026 and pinostrobine) inducers (Figure 1, left panel), that exert their protective effects via disrupting KEAP1-dependent degradation of NRF2, allowing it to translocate to the nucleus and activate transcription of its downstream antioxidant and anti-inflammatory target genes (Jazwa et al., 2011; Morroni et al., 2013; Johnson and Johnson, 2015; Ahuja et al., 2016; Cui et al., 2016; Zhou et al., 2016; Li et al., 2018; Kim et al., 2020). However, an important limitation to the current state of the field is that many of the anti-neurodegenerative effects that have been associated with NRF2 activation were observed in a cell culture setting, or in mouse models where a less severe phenotype is obtained, but the actual levels and form of the compound that pass the BBB and reach affected tissues is unknown. Another caveat is that many covalent inducers are electrophilic, and as such can target reactive cysteines on proteins other than KEAP1, leading to undesirable off-target toxicity that limits their applicability. However, despite this limitation, SF is currently being tested in a phase II clinical trial to determine if it improves cognition in PD patients (Clinicaltrials.gov; NCT05084365), and DMF is approved by the FDA to treat relapsing forms of MS. Along these lines, repurposing DMF to treat PD has garnered some interest (Lastres-Becker et al., 2016), as it is already FDA approved, and it was shown to prevent oxidative stress and cytotoxicity in several *in vitro* and *in vivo* models of PD (Majkutewicz, 2022). Overall, administration of DMF and its other electrophilic counterparts has shown enough benefit to warrant continued development and consideration as alternative approaches are developed and eventually implemented.

Importantly, several alternative strategies have emerged to obtain beneficial induction of NRF2 without using potentially toxic electrophilic compounds. One example is the protein-protein interaction inhibitor (PPI) KKPA4026, which was shown to prevent dopaminergic neuron cell death and ameliorate parkinsonian behavioral deficits in an MPTP model of PD (Kim et al., 2020). Liposomal delivery of resveratrol suppressed oxidative stress and enhanced circulatory function in cerebral vascular cells from aged rats in an NRF2-dependent manner (Csiszar et al., 2015), inferring that improved delivery through the BBB could also enhance the efficacy of other NRF2 inducers. Thus, while electrophilic inducers continue to represent the gold standard, efforts towards improved delivery systems, non-covalent modifiers, and gene-therapy based approaches continue to emerge as more targeted and possibly potent solutions, which will be discussed in more detail below.

#### Alzheimer’s disease

Like PD, several studies have indicated that loss of NRF2 enhances AD phenotypes. For example, amyloid precursor protein/presenilin 1 (APP/PS1) mice lacking NRF2 exhibited autophagy dysfunction-dependent accumulation of insoluble Aβ aggregates, resulting in an increased pro-inflammatory phenotype (Joshi et al., 2015). A similar study testing NRF2 loss in a combined model of amyloidopathy and tauopathy (AT mice) demonstrated that *AT-Nrf2* ^*−/−*^ mice exhibited increased levels of phosphorylated tau, higher levels of Aβ aggregates, and more severe learning and memory deficits than their *AT-Nrf2* ^*+/+*^ counterparts (Rojo et al., 2017). Perhaps one of the more interesting findings from this study was that *Nrf2* ^*−/−*^ mice, even in the absence of excess amyloid or tau, exhibited dysregulation of 7 of the 10 pathways associated with aging and AD progression. This important finding clearly indicates that an age-related decline in NRF2 function is a key driver of neurodegenerative disorders such as AD. Supporting the notion that NRF2 is needed to prevent Aβ-driven AD pathogenesis, mice genetically engineered to overexpress NRF2 in an AD context (*Keap1* ^*FA/FA*^ *;APP* ^*NLGF*^) exhibited increased glutathione, decreased oxidative stress and inflammation, and improved cognition compared to wildtype, with similar benefits being obtained via administration of the isothiocyanate 6-(Methylsulfinyl)hexyl isothiocyanate (6-MSITC) (Uruno et al., 2020).

Also on the pharmacological front, several electrophilic and non-electrophilic compounds have been shown to exert anti-AD effects via activation of NRF2. DMF, SF, and curcumin, much like in a PD setting, have been shown to prevent oxidative stress, inflammation, and pathogenic protein accumulation in *in vitro* and *in vivo* models of AD (Campolo et al., 2018; Paraiso et al., 2018; Xu et al., 2019; Sun et al., 2022). Of note, protection by DMF was observed in male, but not female mice, indicating the possibility of sex-dependent effects on efficacy (Mohle et al., 2021), although further studies to clarify this effect are needed. A pair of non-electrophilic NRF2-KEAP1 PPIs, NXPZ-2 and POZL, discovered by the same group, have both been shown to ameliorate AD phenotypes in Aβ-injected or APP/PS1 mice, respectively (Sun et al., 2020; Sun et al., 2023). One interesting recent alternative to a pharmacological approach is the utilization of antisense oligonucleotides (ASOs) that target the NRF2 machinery. Along these lines, an ASO targeting GSK3-β, which can initiate β-TRCP-dependent degradation of NRF2, was shown to increase NRF2 levels, resulting in decreased oxidative stress and less severe learning and memory impairment in a *SAMP8* ^*−/−*^ AD mouse model (Farr et al., 2014). Thus, much like PD, several pharmacological and non-pharmacological means of targeting the NRF2 pathway have shown therapeutic promise in mitigating AD phenotypes.

#### Huntington’s disease

Unlike AD and PD, where NRF2 localization is altered or its levels are low, no studies, at least to our knowledge, have shown if NRF2 levels are altered in HD patient brains. However, it has been shown that NRF2 is activated in a cell model of huntingtin overexpression, inferring that NRF2 is responsible for mitigating some of the harmful effects brought on by HD progression (van Roon-Mom et al., 2008). Pharmacologically, CDDO-ethyl amide and CDDO-trifluoroethyl amide were shown to decrease oxidative stress and improve motor performance in an N171-82Q transgenic mouse model of HD (Stack et al., 2010). Similarly, naringin, a dietary flavonoid obtained from grapefruit was also shown to activate NRF2-dependent amelioration of HD phenotypes in 3-nitropropionic acid (3-NP)-induced HD (Gopinath and Sudhandiran, 2012). SF, curcumin, and tert-butylhydroquinone (tBHQ) were also shown to protect against 3-NP-induced HD in an NRF2-dependent manner (Sandhir et al., 2014; Jang and Cho, 2016; Silva-Palacios et al., 2017), and a novel covalent modifier MIND4, and its derivative 4–17, activated NRF2 and suppressed oxidative stress in HD cell and animal models, as well as patient monocytes (Quinti et al., 2016; Quinti et al., 2017). Finally, compound 2, a non-covalent chalcone-derived NRF2 inducer, was shown to reduce oxidative stress and improve the survival of H_2_O_2_-treated primary astrocytes isolated from a zQ175 mouse model of HD (Moretti et al., 2021). Overall, NRF2 clearly plays a protective role in preventing HD onset and progression, and efforts continue to determine the relevance of targeting this pathway to treat patients with HD.

#### Other neurological diseases

Two other critical central nervous system disorders, amyotrophic lateral sclerosis (ALS) and multiple sclerosis (MS), have also been shown to involve NRF2 signaling. In the case of ALS, there are contradictory reports indicating that the spinal cord and motor cortex of ALS patients have both lower and higher mRNA and protein levels of NRF2 (Sarlette et al., 2008; Lastres-Becker et al., 2022). However, the study by Lastres-Becker et al. also tested downstream target genes, showing elevated expression of the key detoxifying target gene NADPH-quinone oxidoreductase 1 (*NQO1*), as well as the iron metabolism protein heme-oxygenase 1 (*HMOX1*). Thus, while this study infers target gene activation does occur in ALS tissues, further clarification of NRF2 levels in ALS patients is needed. Much like the MPTP-induced model of PD, the cell type-relevance of NRF2 may also be important to consider, as astrocyte-specific overexpression of NRF2 increased survival of mice overexpressing mutant superoxide dismutase 1 (*SOD1* ^*G39A*^), an established model of ALS (Vargas et al., 2008). Conversely, neither whole body knockout, nor targeted overexpression of NRF2 in neurons or skeletal muscle had a pronounced effect on *SOD1* ^*G39A*^ mouse survival (Guo et al., 2013; Vargas et al., 2013). This was further supported by a later study, which investigated a possible gene therapy-based approach via adeno-associated viral delivery of *NFE2L2/*NRF2*,* which was able to activate NRF2 and its downstream genes *NQO1* and *HMOX1* in NSC-24 motor neuron cells and *SOD1* ^*G39A*^ mice; however, overall mouse survival was unaffected (Nanou et al., 2013). These studies further highlight the cell type-relevance of NRF2 in different neurodegenerative contexts, and that additional experimental models of ALS may need to be considered to better correlate patient observations with lab-based studies.

Finally, NRF2 has also shown importance in MS models and patient contexts. Like ALS, NRF2 was shown to be upregulated in MS patient lesions (Licht-Mayer et al., 2015), and transgenic activation of NRF2 specifically in astrocytes prevented, whereas whole body knockout exacerbated, the oligodendrocyte loss and enhanced inflammation observed in a cuprizone-induced model of MS (Draheim et al., 2016; Nellessen et al., 2020). Mentioned briefly above, the gold standard treatment for MS, DMF (Tecfidera), has also been shown to activate NRF2 in MS patient blood and immune cells (Gopal et al., 2017; Hammer et al., 2018; Carlstrom et al., 2019). Furthermore, DMF-dependent activation of NRF2 in neurons and glia was associated with decreased oxidative stress and increased overall survival in a myelin oligodendrocyte glycoprotein-driven mouse model of MS. The protective effect of DMF was not observed in *Nrf2* ^*−/−*^ mice, although a later study using this same model produced contradictory results, which could be due to discrepancies in DMF dose and time of treatment (Linker et al., 2011; Schulze-Topphoff et al., 2016). In general, these studies indicate that activation of NRF2 represents a feasible strategy to treat MS progression, with DMF and its derivatives representing the best current approach.

#### Caveats and future considerations

**FIGURE 2:**
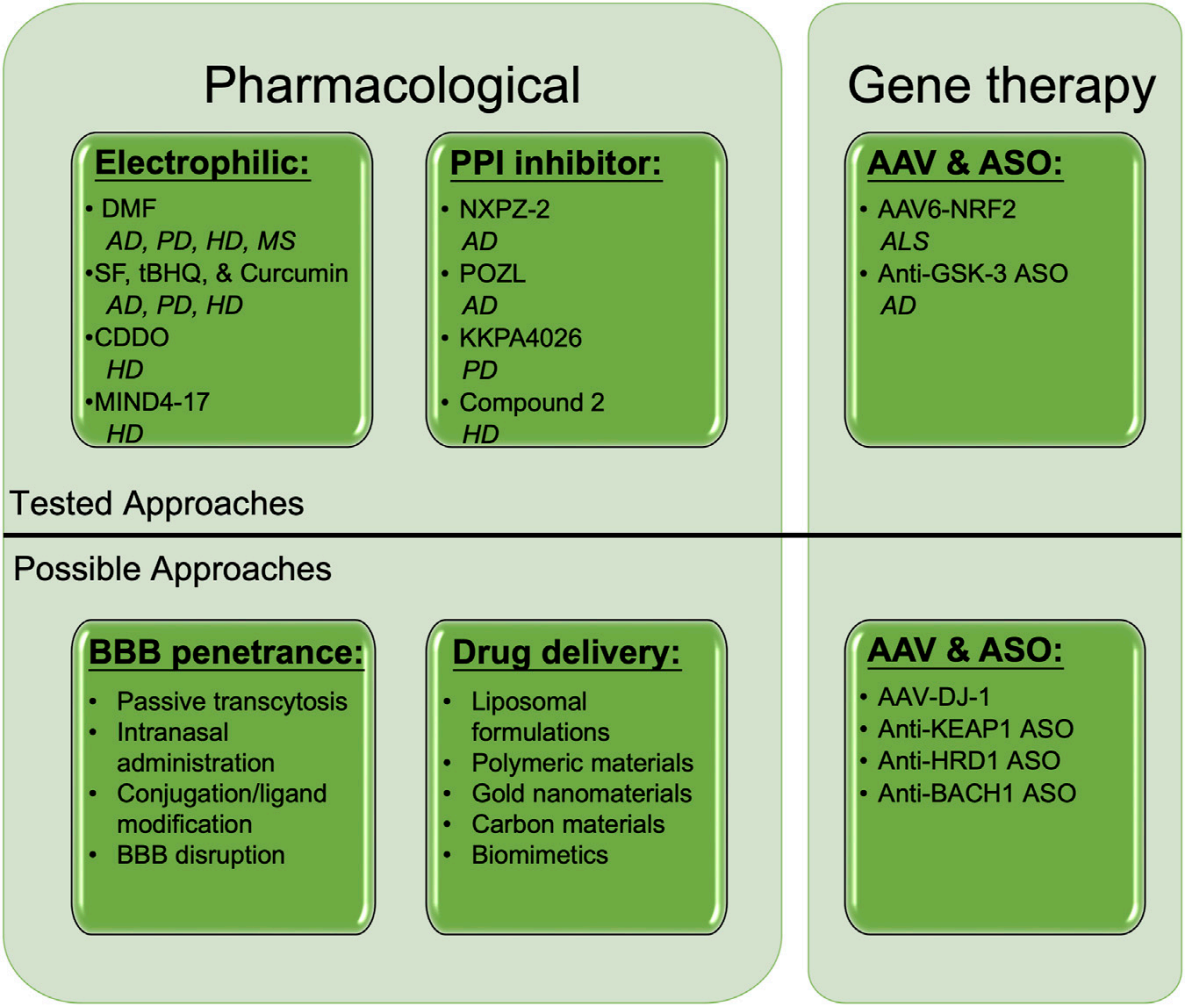
Established and putative means of NRF2 activation to treat neurodegeneration. Several pharmacological and gene therapy-based approaches have shown therapeutic promise in treating neurodegenerative diseases. Electrophilic activators, including DMF, SF, tBHQ, Curcumin, CDDO, and MIND4-17, as well as non-covalent protein-protein interaction inhibitors such as NXPZ-2, POZL, KKPA4026, and Compound 2 have all been shown to ameliorate AD, PD, HD, and MS progression in an NRF2-dependent manner. Adenovirus-associated NRF2 (AAV6-NRF2), as well as antisense oligonucleotides (ASOs) against GSK-3β, have been shown to prevent ALS and AD phenotypes, respectively. Possible untested pharmacological, drug delivery, and AAV/ASO approaches to target NRF2 in neurodegenerative disease include passive transcytosis, intranasal delivery, drug modification, membrane disruption, liposomal/nanoparticle formulations, and AAV/ASOs targeting NRF2 stabilizers (DJ-1), or its degradation machinery (KEAP1, HRD1) and transcriptional repressor (BACH1). DMF = Dimethyl fumarate, SF = Sulforaphane, tBHQ = tert-butylhydroquinone, BBB = Blood brain barrier, AAV = Adeno-associated virus, ASO = Antisense oligonucleotide. Created with Biorender.

Activation of NRF2 continues to represent a therapeutic strategy with promising, yet untapped potential. As loss of NRF2 clearly exacerbates the progression of experimental models of neurodegenerative disorders, it remains clear that preserving or re-establishing proper NRF2 function should mitigate disease progression and improve patient prognosis across a wide range of neuropathological contexts. While the most promising options identified to date are electrophilic (i.e., SF, DMF, and CDDO), alternative approaches, including protein-protein interaction inhibitors, adeno-associated viral (AAV)-mediated delivery, antisense oligonucleotides, and enhanced delivery systems continue to emerge as viable possibilities (Figure 2). Another interesting approach currently being tested is hybrid molecules, whereby activators of NRF2 (i.e., DMF) are coupled to molecules that inhibit its upstream repressors or co-activate its downstream effectors (i.e., GSK3- β and HMOX1) (Di Martino et al., 2020; El Ali et al., 2020). However, as discussed above, continued reliance on electrophilic compounds with known off-target effects is still likely to result in toxicity regardless of the specificity of the conjoined molecule. Another intriguing class of compounds not discussed in detail here is natural compounds (Moratilla-Rivera et al., 2023), which clearly exert beneficial effects, but often lack sufficient clarity on the mechanism of action and whether the compound itself, or a metabolite, are responsible for the observed protection. Regardless, pharmacological intervention continues to warrant further investigation, particularly in cases where no toxicity is observed.

Along with the methods already being tested in an NRF2 context, several other possible strategies for therapeutic intervention also warrant consideration. One popular approach is improving the ability of small molecules to cross the BBB. This includes mildly disrupting BBB integrity, modifying/tagging established compounds to improve their stability/penetrance, as well as utilizing intranasal administration to bypass the BBB altogether, among others (Figure 2). Much like the liposomal delivery approach discussed above, several nanoparticle-, bioengineering-, and biomimetic-based approaches have also garnered recent interest (Sun and Roy, 2021), inferring that testing these systems with NRF2-targeted therapies could also work. Finally, based on the promise of AAV-mediated NRF2 overexpression, as well as targeted enhancement of NRF2 transcription in astrocytes in mouse models of neurodegeneration, gene therapy-based approaches that lead to brain cell type-dependent increases in NRF2 expression also appear to have significant merit. This is further supported by the beneficial, NRF2-dependent, effects observed in the presence of ASOs targeting GSK3-β, as other ASOs targeting negative regulators of NRF2 signaling (i.e., KEAP1, synoviolin [HRD1], and the transcriptional repressor or NRF2-AREs, BACH1, could all theoretically upregulate NRF2 to provide therapeutic benefit (Figure 2). AAV-mediated overexpression of DJ-1, which has been shown to stabilize NRF2 in a PD context (Clements et al., 2006; Im et al., 2012), could also be effective. Continued testing of these and other already established NRF2-based strategies promises to yield better NRF2-targeted therapies that progress to clinical trials and can eventually be used for intervention in patients suffering from these debilitating diseases.

## Concluding remarks

NRF2 continues to represent a viable therapeutic target with endless possibilities. While current efforts have shown great promise, the field has continued to evolve towards more targeted, efficient, and potent possibilities. Considering the NRF2 field is still relatively young, at just over two decades old, the progress made to date regarding our mechanistic understanding of this pathway in disease, including viable means to target it even at the patient level, is remarkable. Clearly the sky is the limit in harnessing the protective potential of this pathway across the neurodegenerative disease spectrum, and only time will tell if we can finally progress from experimental promise to therapeutic reality.

## Data Availability

The original contributions presented in the study are included in the article/supplementary material, further inquiries can be directed to the corresponding author.
